# New insights into the standard method of assessing bacterial filtration efficiency of medical face masks

**DOI:** 10.1038/s41598-021-85327-x

**Published:** 2021-03-15

**Authors:** Jérémie Pourchez, Aurélien Peyron, Yoann Montigaud, Coralie Laurent, Estelle Audoux, Lara Leclerc, Paul O. Verhoeven

**Affiliations:** 1 Mines Saint-Etienne, Univ Lyon, Univ Jean Monnet, INSERM, U 1059 Sainbiose, Centre CIS, 42023 Saint-Etienne, France; 2 CIRI (Centre International de Recherche en Infectiologie), Equipe GIMAP (team 15), INSERM U1111, CNRS, ENS, UCBL1, Université Jean Monnet, Université de Lyon, Saint-Etienne, France; 3 Service des Agents Infectieux et d’Hygiène, CHU de St-Etienne, Saint-Etienne, France; 4 École Nationale Supérieure Des Mines Saint Étienne, INSERM, U 1059 Sainbiose Centre CIS, 42023 Saint-Etienne, France

**Keywords:** Bacteria, Microbiology techniques, Biomedical engineering, Biological physics

## Abstract

Based on the current knowledge of severe acute respiratory syndrome coronavirus-2 (SARS-CoV-2) transmission, wearing a mask has been recommended during the COVID-19 pandemic. Bacterial filtration efficiency (BFE) measurements enable designing and regulating medical masks to prevent bioaerosol dissemination; however, despite the simplicity of these measurements, several scientific questions remain unanswered regarding BFE tests. Here, we investigated (1) the impact of substituting 100-mm Petri dishes with 90-mm disposable Petri dishes, (2) the impact of colony-counting methods on the bioaerosol aerodynamic size, and (3) the impact of colony-counting methods on the total viable particle counts. We demonstrated that disposable 90-mm Petri dishes can be used to replace the 100-mm dishes. We also showed that an automatic high-resolution colony counter can be used to directly count viable particles on collection substrates and to measure the bioaerosol size parameters. Our results enable possible modernization of the outdated testing methods recommended in the US and European standards for BFE measurements. Specifically, use of a modernized colony counter should be clearly regulated and permitted to avoid the counting of positive holes. The median aerodynamic diameter appears to be the most relevant parameter for characterizing bioaerosol size.

## Introduction

Transmission routes of severe acute respiratory syndrome coronavirus-2 (SARS-CoV-2) include^1,2^ (1) contact transmission (i.e., direct contact with an infectious person or contaminated surface), (2) droplet transmission (i.e., virus-containing respiratory droplets emitted by sneezing or coughing), and (3) airborne transmission (i.e., infectious particles produced by desiccation of larger droplets in the airstream). For some viral and bacterial pathogens, airborne transmission appears to be a major mode by which people are infected, as is the case with *Mycobacterium tuberculosis* (the bacterium that causes tuberculosis), rubeola (the virus that causes measles), and Varicella-Zoster (the virus that causes chicken pox). Although these diseases can be transmitted at close ranges, they are efficiently and frequently transmitted to people passing through a room in which an infectious person was present minutes to hours earlier. Conversely, the SARS-CoV-2 epidemiology suggests that infections are mainly transmitted via close contact (i.e., contact or droplet transmission), although they can sometimes be spread via airborne transmission under special circumstances^1^. Several well-documented examples have shown uncommon transmission events of SARS-CoV-2 over long distances or times when an infectious person produced respiratory droplets for an extended time in an enclosed space^3–5^. Thus, as recommended against most respiratory infections, current COVID-19 infection control policies aim to limit people’s exposure to infectious aerosols that can span a wide range of aerodynamic sizes from larger respiratory droplets that fall out of the air within seconds to minutes and remain near the infectious person, to smaller particles suspended in the air over long distances for several hours.

Owing to the current knowledge of SARS-CoV-2 transmission, wearing a mask has become commonplace during the COVID-19 pandemic. Accumulating evidence shows that face masks can help prevent SARS-CoV-2 transmission^6^. Masks are indicated for protecting the wearer and their environment and for preventing exhalation of potentially infectious respiratory droplets into the airstream. Many data demonstrate that wearing masks helps effectively reduce spread of the virus via respiratory secretions (i.e., the source control strategy) and helps protect people who wear them correctly from contracting COVID-19^6,7^. A meta-analysis of 21 studies concluded that masks provide significant protection and reduce transmission cases among non-healthcare workers by 47%^8^. Worby and Chang demonstrated that public use of face masks can effectively mitigate SARS-CoV-2 transmission in multiple scenarios^9^. Finally, a recent systematic review^10^ of 172 observational studies in 16 countries and on six continents without randomized trials and 44 relevant comparative studies in healthcare and non-healthcare settings (n = 25,697 patients) showed that using face masks significantly reduced the risk of infection. Wearing any type of face mask decreases the infection risk by fivefold from 17.4 to 3.1%^10^.

Among the different categories of face masks, medical masks are the only masks specifically designed and regulated to prevent bioaerosol dissemination from the wearer into the environment (i.e., aerosol from the upper airways or saliva that may contain infectious agents transmissible by droplet or airborne routes). Medical face masks are classified as medical devices with an official standard description (EN 14683:2019^11^ in Europe or ASTM F2100-19^12^ in the US). Masks specified under the EN 14683:2019 standard are classified into two types according to their bacterial filtration efficiency (BFE) level. Type I masks have a BFE ≥ 95%; type II masks have a BFE ≥ 98%. Masks that meet the level 1 and level 2 requirements of the ASTM F2100-19 standard are equivalent to type I and type II masks, respectively, of the EN 14683:2019 standard. Both standards^11,12^ are very similar in their final mask specifications and test methods for assessing mask performance; therefore, we retained the terminologies used in the EN 14683:2019 standard throughout this manuscript for consistency. In addition to the BFE measurement, the performance requirements of medical face masks include a microbial cleanliness (i.e., the bioburden of the mask) < 30 colony-forming units (CFUs)/g, breathability (i.e., the air permeability of the mask determined from the differential pressure across the mask) < 40 Pa/cm^2^ (for types I and II) or 60 Pa/cm^2^ (for type IIR), and a splash resistance for type IIR masks (i.e., resistance of the mask to penetration from splashing) > 16.0 kPa.

The BFE is a critical parameter for determining medical mask performance. The standard test methods for assessing the BFE are similar for both the EN 14683:2019 and ASTM F2100-19. Briefly, a sample of the mask material is clamped between a six-stage viable Cascade Impactor and an aerosol chamber. A bioaerosol containing *Staphylococcus aureus* is generated at the top of the aerosol chamber, then 3-µm droplets that can contain a 1-µm bacterium are introduced into the aerosol chamber and drawn through the mask material and the impactor under vacuum. The BFE is equal to the number of CFUs passing through the mask material and is expressed as a percentage of the CFUs present in the challenge aerosol. However, the simplicity of the BFE test raises several unanswered scientific questions. The six-stage viable Andersen cascade impactor (ACI) is a multiple-jet impactor with 400 holes that collect aerosols containing live bacteria at each stage. The air jets impinge directly onto nutrient agar in a Petri dish, which is incubated after sampling until the collected bacteria multiply into colonies. However, the resulting bacterial counts on the Petri dish can be inexact, and the observed number of colonies can be adjusted for the likelihood that more than one viable particle was collected through a sampling hole and merged with other bacteria at an impaction site to produce a single colony, although several bacteria passed through the same hole during the test. Hence, more than 3 decades ago, a “positive-hole” correction table was proposed to correct the positive-hole visual counts obtained using viable cascade impactors^13^. In 2020, this methodology seems archaic. Compared with the time-consuming, operator-dependent, visual counting of the positive holes on Petri dishes, which can lead to sources of bias, and their conversion into “viable particles”, direct colony counting using high-resolution (HD) automatic color colony counters ensures greater user comfort and higher accuracy and reproducibility in directly counting the total number of bacteria collected on Petri dishes, potentially without requiring corrections.

For decades, before the COVID-19 pandemic, only a few private for-profit laboratories worldwide conducted regulatory tests on masks for medical use, particularly the BFE test, which has undergone few changes since its initiation. The mask shortage at the start of 2020 revealed the need to increase the availability of face masks and to increase the number of expertise centers capable of performing regulatory tests to measure mask performance. Our academic laboratory was the first in France to be accredited by the French National Agency for Medicines and Health Products Safety (ANSM) to perform the BFE standard test for the duration of the ongoing COVID-19 pandemic. With several months of experience and several hundred masks tested, our research laboratory has undertaken studies to better understand the scientific issues behind the BFE regulatory test to modernize the way this standard test is conducted. Consequently, we assessed two methods for assessing bacterial counts from a BFE test: direct colony counting using an HD automatic colony counter with or without conversion into viable particles and the gold standard procedure (the standard procedure) of “corrected particle counting from positive-hole conversion” (i.e., visual, nonautomated counting of positive holes, then calculating the number of viable particles using a conversion table). The impact of these colony-counting methods was determined on the total number of bacteria aerosolized by a bioaerosol nebulizer and on the median and mean aerosol particle sizes. We also examined the impact of a change in Petri dish sizes introduced into the six-stage viable ACI.

## Materials and methods

### Aerodynamic particle sizing using a six-stage viable ACI

The six-stage viable ACI (Tisch Environmental, Cleves, OH, USA) is a multi-orifice cascade impactor requiring an exact flow rate of 28.3 L/min using a vacuum pump. Each impactor stage contains up to 400 precision machine jet orifices (i.e., “holes”). The size of the jet orifices is constant within each stage but becomes smaller in each succeeding stage with diameters ranging from 1.81 mm in the first stage to 0.25 mm in the sixth stage. The range of particle sizes collected in each stage depends on the jet velocity of the stage and the cut-off of the previous stage. The 50% effective cut-off diameters (i.e., the particle diameters corresponding to 50% sampling efficiency) for each of the six stages when operating at 28.3 L/min are 7 µm (stage 1, with orifices of 1.81 mm in diameter), 4.7 µm, 3.3 µm, 2.1 µm, 1.1 µm and 0.65 µm (stage 6, with orifices of 0.25 mm in diameter).

**Figure 1:**
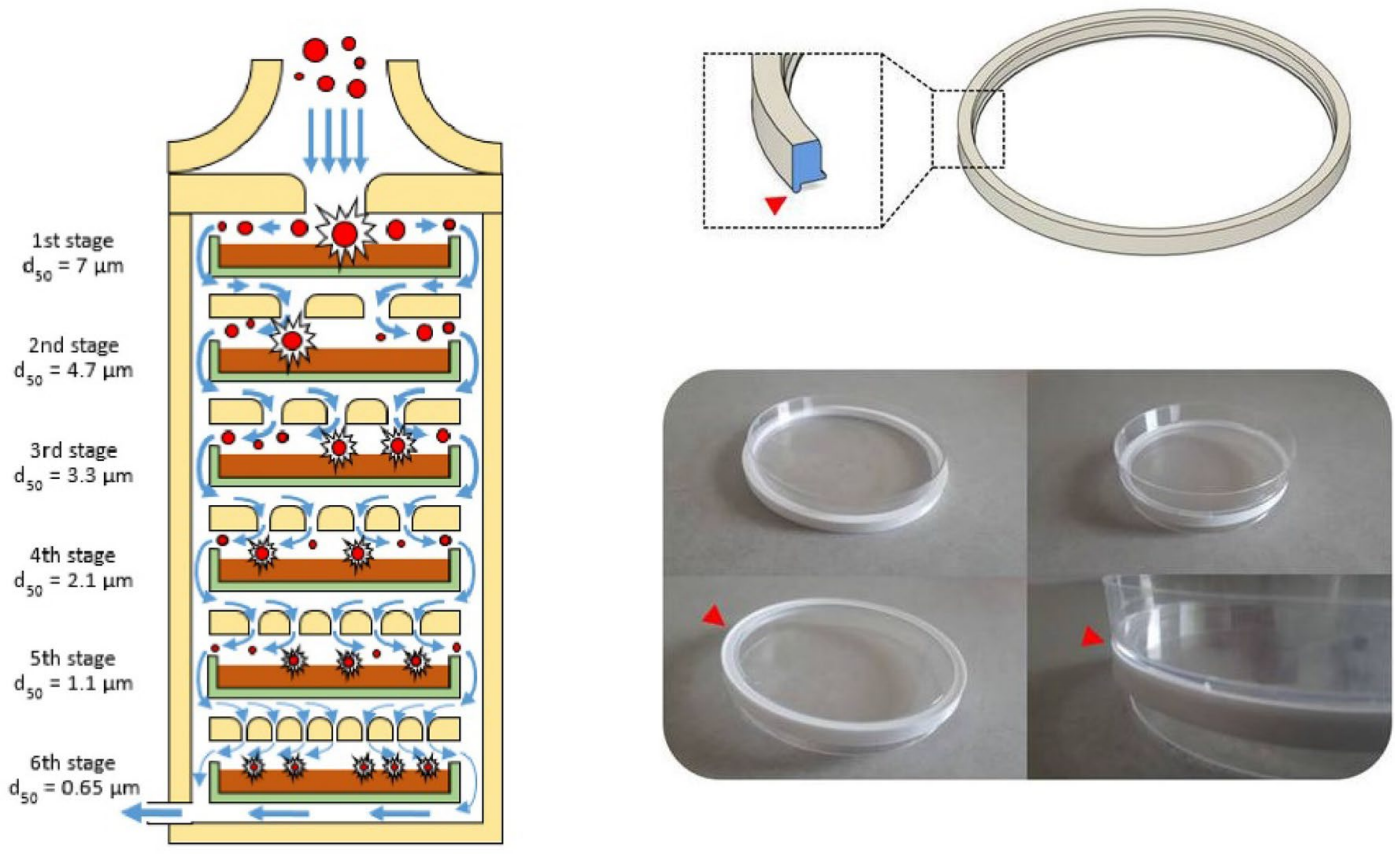
(Left) Schematic of a six-stage viable ACI (yellow). Airborne particles (red) are aspirated inside the cascade impactor with a 28.3 L/min flow rate (blue arrow). Each stage of the ACI contains a Petri dish (green) filled with nutrient agar (brown). (Right) Overview of the 3D-printed adaptors to insert the commercial reference 90-mm plastic Petri dishes inside the ACI instead of the specific glass Petri dishes supplied with the ACI.

The six-stage viable ACI is frequently used to measure the concentration and particle size distribution of airborne bacteria. This aerosol sizing instrument allows enumerating the viable particles in a microbial aerosol. This sampler was calibrated so that all collected particles, regardless of physical size, shape, or density, are sized aerodynamically and can be directly related to human lung deposition. Petri dishes are positioned inside the cascade impactor to directly collect the airborne bacteria. When air is drawn through the sampler, multiple jets of air in each stage direct airborne particles of given aerodynamic sizes toward the surface of the Petri dish collecting the particles at that stage (Fig. 1). Any particle not collected in stage 1 follows the air stream around the edge of the Petri dish to the next stage. Viable particles collected on Petri dishes are then incubated for colony counting and identification.

### Substituting 100-mm glass Petri dishes with 90-mm plastic Petri dishes inside the ACI

The instruction manual of the six-stage viable ACI states that only the glass Petri dishes supplied with the instrument should be used after their sterilization. Using Petri dishes other than those supplied is contraindicated because this would alter the distance between the jet orifice and the collection surface at each stage. Additionally, plastic Petri dishes should not be used because they generate static charges that reduce the collection efficiency. When substituting the specific 100-mm glass Petri dishes with a commercial reference of 90-mm disposable plastic Petri dishes, validation of the impact on aerosol particle sizing and bacterial collection must be critically examined. After testing nearly a dozen commercial references, we herein present only the results obtained from the plastic Petri dishes (VWR REF 391-0601, France) to allow obtaining the best substitution results. To prevent movement of the 90-mm Petri dishes inside the ACI when the vacuum pump causes a 28.3 L/min flow rate, three-dimensional (3D)-printed adaptors were designed to be placed on each stage (Fig. 1).

### Positive-hole conversion into viable particles

The instruction manual of the six-stage viable ACI recommends that only agar plates containing > 300 colonies be counted via the positive-hole method. This document also states that the positive-hole method is rarely used today and is less accurate than visually counting colonies. However, as discussed in the next subsection on standard BFE testing, the US and European standards still impose the use of this counting method for stages 3–6 during BFE testing although imaging-based automated colony counter are able to reliably quantify plate with higher bacterial load.

**Figure 2:**
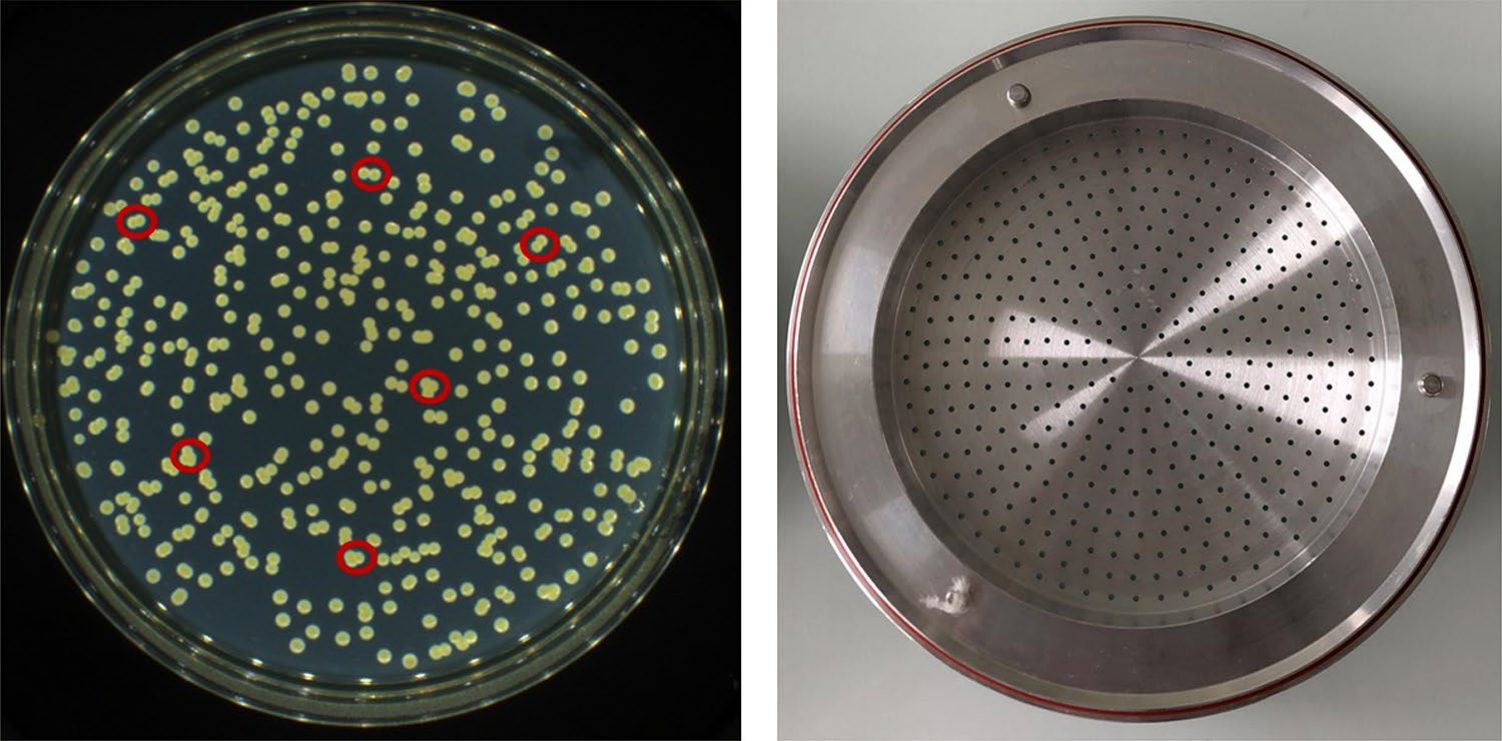
(Left) colony observation using an HD colony counter on a Petri dish from stage 3 of a positive BFE test run, inside the red circle cluster of colonies from the same impaction site showing several individual colonies from the same hole easily counted by a modern HD colony counter. (Right) ACI stage with the 400 precision machined jet orifices.

As a function of the counting methodology used, the observed number of colonies on the Petri dish may not correspond to the number of viable particles impacted on the agar collection surface. A bacterial colony forms at each impaction site where one or more viable particles coming from only one jet or hole were deposited (Fig. 2). Hence, the observed number of colonies can be adjusted for the likelihood that more than one viable particle was collected through a sampling hole and merged with other bacteria at an impaction site to finally produce a single colony, although several bacteria passed through the same hole during the test.

Thus, the positive-hole method essentially involves counting the jets that delivered viable particles to the Petri dishes, then converts this count to a “viable particle” count using the positive-hole conversion table (Supplementary Table 1) proposed by Macher in 1989^13^. This table is based on the principle that as the number of viable particles being impinged on a given plate increases, the probability of the next particle going into an empty hole decreases. For example, when 9/10 of the holes have received one or more particles, the next particle has only a one in ten chance of going into an empty hole. Thus, at this point, an average of ten additional particles would be required to increase the number of positive holes by one, and before all the holes become positive, some holes will receive multiple particles.

Using the positive-hole conversion table, the number of positive holes must be precisely determined. A colony out of the hole pattern is not counted as a positive hole. However, this step is inexact owing to the difficulty in determining whether each colony observed is in or out of the same hole pattern (Fig. 2). Furthermore, this visual counting technique is highly time-consuming and operator-dependent. In 2020, the use of a modern optical and automatic HD colony counter would allow directly counting the viable particles because several colonies can be easily observed at each impaction site coming from the same hole. Nevertheless, some of the colonies observed when using the HD colony counter may exhibit perfect colocalization of several viable particles from the same hole. Hence, researchers much determine whether the colony counts obtained using a modern HD optical counter for low incubation times (to obtain the smallest colonies) correspond more strongly to a positive-hole count (thus requiring conversion into viable particles) or to a direct count of the viable particles.

### Standard BFE testing

**Figure 3:**
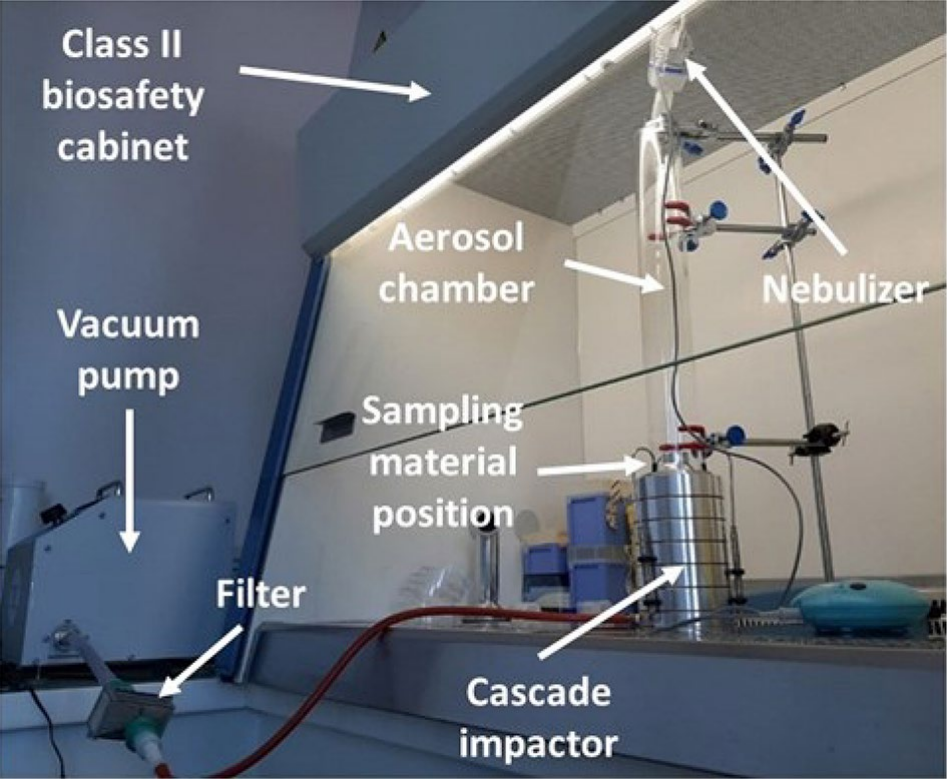
BFE test apparatus with the experimental bench developed in March 2020 and certified by the French National Agency for Medicines and Health Products Safety (ANSM) as compliant with the EN 14683:2019 standard test method.

BFE testing was assessed following the EN 14683:2019 standard devoted to surgical mask performance. Figure 3 shows the BFE test apparatus. Briefly, a sample of the mask material is clamped between a six-stage viable ACI and an aerosol chamber (glass, 445 mm long and 60 mm in external diameter). To perform the BFE experiments in a class II biosafety cabinet, we reduced the length of the aerosol chamber in our experimental set-up (600 mm long and 80 mm in external diameter according to EN 14683:2019) without changing the features of the bioaerosol arriving at the top of the ACI. Test samples with a minimum size of 100 mm by 100 mm and that included all mask layers were cut from complete face masks. Each test specimen was conditioned at 21 ± 5 °C and 85 ± 5% relative humidity for the time required (at least 4 h) to reach atmospheric equilibrium prior to testing. At least five samples were tested. The testing was performed with the inside of the medical face mask in contact with the airborne bacteria.

*Staphylococcus aureus* (ATCC 6538) was inoculated into 30 mL of tryptic soy broth in an Erlenmeyer flask and incubated with mild shaking at 37 ± 2 °C for 24 ± 2 h. An aerosol containing *S. aureus* was introduced into the aerosol chamber and drawn through the mask material and the impactor under vacuum. The choice of bioaerosol nebulizer is critical. The EN 14683 imposes only two specifications: a mean particle size (MPS) of 3.0 ± 0.3 µm and 1700–3000 CFUs of bacteria per test. The viable particle count values used for the MPS calculations (Eq. 1) were converted to “probable hit” counts calculated using the positive-hole conversion chart from the cascade impactor manual. The bacterial challenge corresponded to the viable particle counts observed on the sixth positive-control dishes.

1 $$MPS= \frac{\left(P1\times C1\right)+\left(P2\times C2\right)+\left(P3\times C3\right)+\left(P4\times C4\right)+\left(P5\times C5\right)+\left(P6\times C6\right)}{C1+C2+C3+C4+C5+C6}$$

Calculation of the mean particle size (MPS) using Px (the 50% effective cut-off diameters of each of the six stages, x = [1–6]) and Cx (the viable particle counts obtained from each of the six agar collection surfaces, x = [1–6]).

Recent studies on bioaerosol generation^14–18^ used a modern vibrating mesh nebulizer as an alternative to older jet nebulizers (such as the collision jet) that have been used for decades to perform BFE tests. Consequently, the E-Flow mesh nebulizer (Pari GmbH, Starnberg, Germany) provided aerosolization of 3.0 ± 0.3 µm droplets from a 3-mL suspension with 3000 CFU/mL of *S. aureus*. A bacterial challenge of 1700–3000 CFUs per test was maintained with a 1-min nebulization duration. The airflow was maintained through the cascade impactor for 1 additional minute (the total test time was thus 2 min). Petri dishes were removed from the ACI and incubated at 37 ± 2 °C for 22 ± 2 h before counting colonies either with an automatic HD colony counter (Scan 1200, Intersciences, France) or by visually counting the positive holes and converting them into viable particles. The colony counter used is a high resolution automatic color colony counter with automatic lighting with the given specifications: minimum colony size: 0.05 mm, 1000 colonies CFU detected in 1 s, Live image using zoom × 28, Counts 30 dishes in 5 min (in real conditions with presetting), fully automatic lighting (lights controlled by computer and motorized background color), resolution of 1280 × 960 pixels, excellent reproducibility and repeatability (Good Laboratory Practice, CFR 21 Part 11).To evaluate the BFE of a mask, a series of eight successive measurements must be performed. First, a positive-control run is performed without a mask positioned between the cascade impactor and aerosol chamber. Next, five experiments are performed on test samples, changing the mask for each experiment and cleaning the experimental set-up to avoid bacterial contamination. A second positive control experiment is then performed. Finally, this cycle of eight consecutive experiments ends with a negative-control run in which air is passed, without adding bacteria, through the cascade impactor for 2 min (this serves as a contamination control to verify that the bacteria deposited during the positive run and the test samples came only from the bioaerosol source).

The BFE of the mask is equal to the number of CFUs passing through the medical face mask material expressed as a percentage of the number of CFUs present in the challenge aerosol (from the positive control runs performed with no testing material between the cascade impactor and the aerosol chamber). The BFE was calculated using Eq. (2):

2 $$\mathrm{B}=\frac{\mathrm{C}-\mathrm{T}}{\mathrm{C }\times 100}$$

Calculation of the BFE (B is expressed as a %), where C is the mean of the two positive runs of the total of the six plate counts, and T is the total of the six plate counts for each test sample.

### Colony-counting methods

For all experiments described herein, we used large amounts of airborne bacteria collected by the ACI. Thus, we used only positive-control runs with the BFE EN 14683:2019 standard test method, i.e., experiments without testing material between the cascade impactor and aerosol chamber.

**Figure 4:**
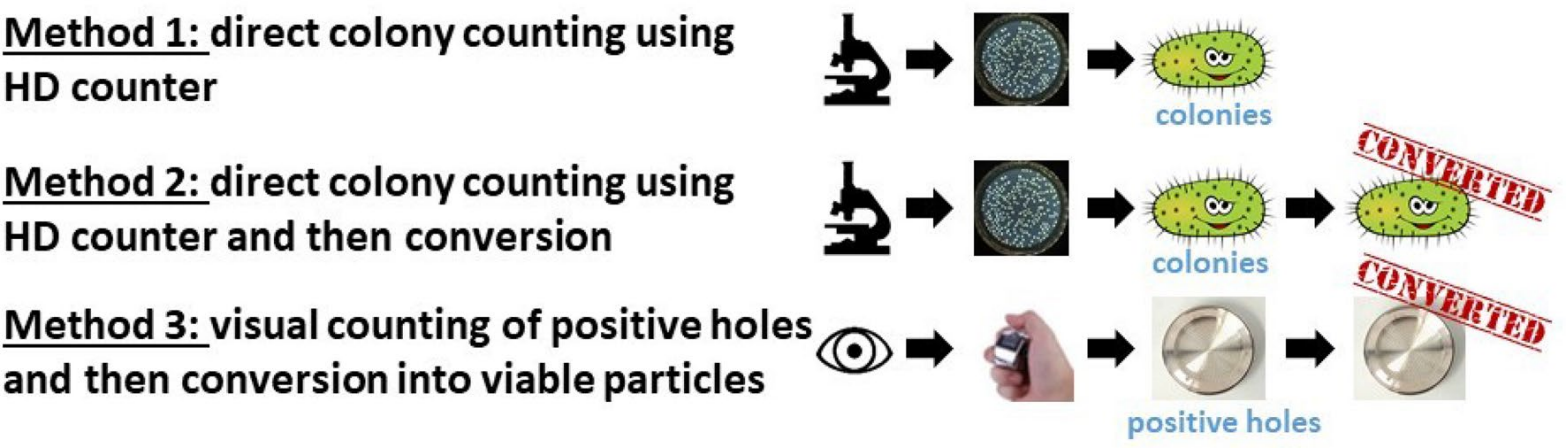
Colony-counting methods used in this study.

Because a major point of this study was to identify whether using automatic HD colony counter results in a direct counting of viable particles or a direct counting of positive holes, the two methods (methods 1 and 2, Fig. 4) were used to interpret each positive-control run of the BFE standard test method. The gold standard method recommended by EN 14683:2019, i.e., visually counting the positive holes and converting them into viable particles, was used as a reference (method 3, Fig. 4).

An additional method allowed approximating the bacteria delivered by the bioaerosol nebulizer. The total airborne bacteria emitted by the nebulizer were accurately quantified for each experiment using the bacterial suspension in CFU/mL initially introduced into the nebulizer tank (using an easy SPIRALE dilution and inoculating 50 µL of our bacterial preparation onto Columbia agar + 5% sheep blood) and the weight that was lost from the suspension during the 1 min aerosolization in the bacterial challenge. Except for the bacteria lost on the aerosol chamber surface, this calculation is a good approximation for use as a control in this study compared with the mean of the total of the six plate counts for a positive run (C in Eq. 2).

### Statistical analysis

Statistical analyses were performed with GraphPad Prism 8.4.2 (GraphPad Software, San Diego, CA, USA). Unless otherwise stated, data are presented as the mean ± standard deviation. The three described methods were compared using a repeated-measures 2-way analysis of variance followed by either Sidak’s multiple-comparison post hoc test or Tukey’s multiple-comparison post hoc test. A Gaussian distribution was assumed for all data except for the distributions that were tested for normality with a Shapiro–Wilk’s test, skewness and kurtosis.

## Results

### Effect of the colony-counting methods on the total bacteria/viable particle counts

We described the results obtained from one plate from a positive BFE run to qualitatively illustrate the colony-counting methods considering the Petri dish corresponding to stage 3 of the ACI in Fig. 2. Using method 1, we observed 479 colonies with the automatic HD counter (considering these 479 colonies as viable particles, they should be equivalent to 279 positive holes as per the conversion table). Using method 2, we observed 584 viable particles (the result of converting the 479 colonies observed using the automatic HD counter if the 479 counts are considered positive holes). Using method 3, we visually counted 307 positive holes corresponding to 584 viable particles after conversion. Thus, the automatic HD colony counter allowed counting > 400 colonies on the plates (e.g., 479 counts in this example).

**Figure 5:**
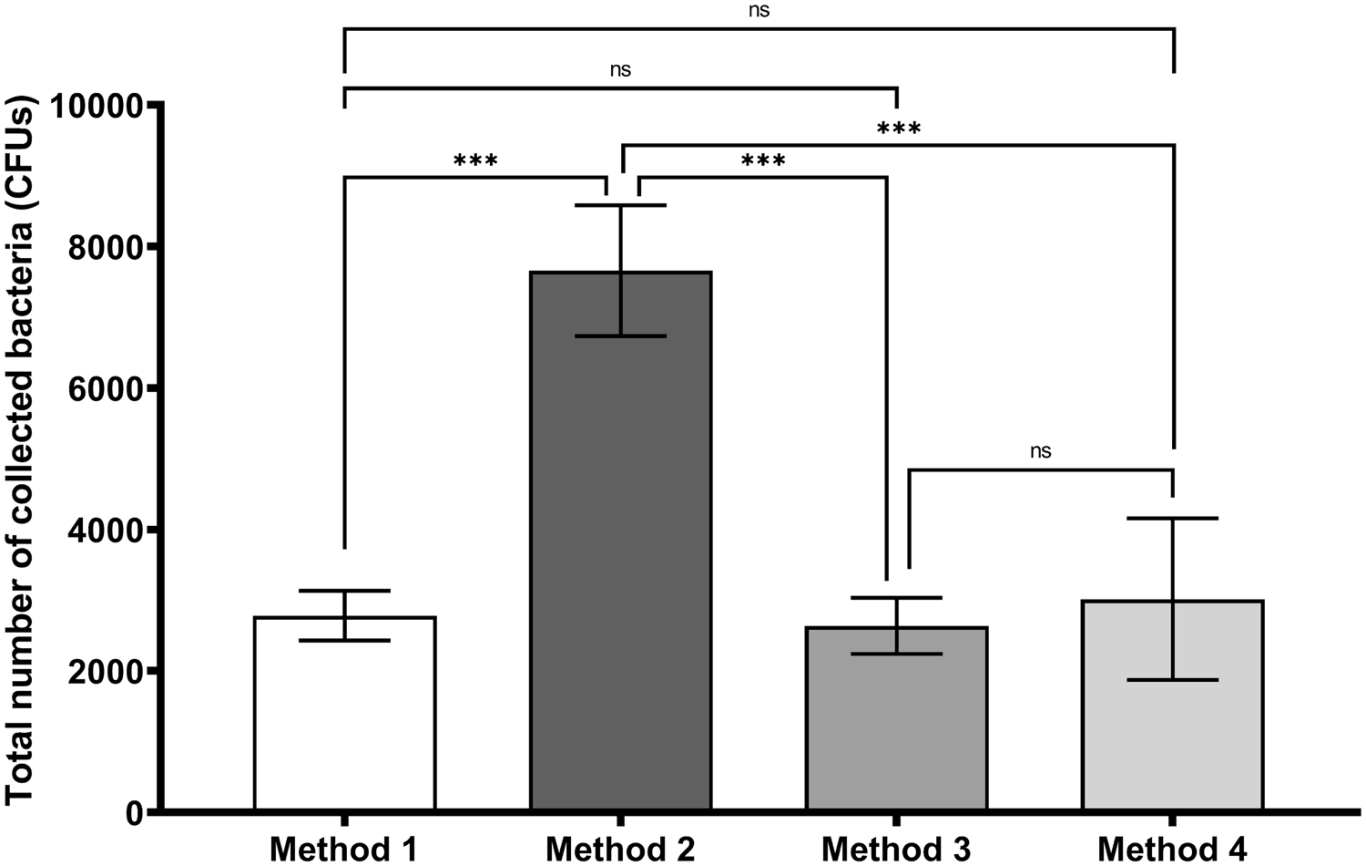
Impact of colony-counting methods on the total number of collected bacteria in the six stages of the ACI for positive BFE runs (n = 4 for two different bacterial suspensions; 8 nebulizations/100-mm Petri dish according to the ACI instruction manual). Method 1 corresponds to direct colony counting using an HD counter (2781 ± 460 counts). Method 2 corresponds to direct colony counting using an HD counter and converting the count into viable particles (7659 ± 1177 counts). Method 3 corresponds to visual colony counting of positive holes and then conversion (2638 ± 480 counts). Method 4 was used as a reference, corresponding to the approximation of the total airborne bacteria emitted by the bioaerosol nebulizer (3014 ± 1144 counts). ns: not significant; *p < 0.05; **p < 0.01; ***p < 0.001.

For a quantitative analysis, we performed eight positive BFE runs (corresponding to 48 Petri dishes to be counted because six plates were analyzed in positive runs). The results from methods 1 and 3 did not statistically differ, while method 2 resulted in greatly overestimating the colony counts (Fig. 5). Method 4 was used as a reference to estimate the total airborne bacteria emitted by the bioaerosol nebulizer. A nonstatistical difference was observed between methods 1 and 3 and reference method 4.

### Impact of substituting the 100-mm Petri dishes with 90-mm disposable Petri dishes on the total bacteria/viable particle counts

**Figure 6:**
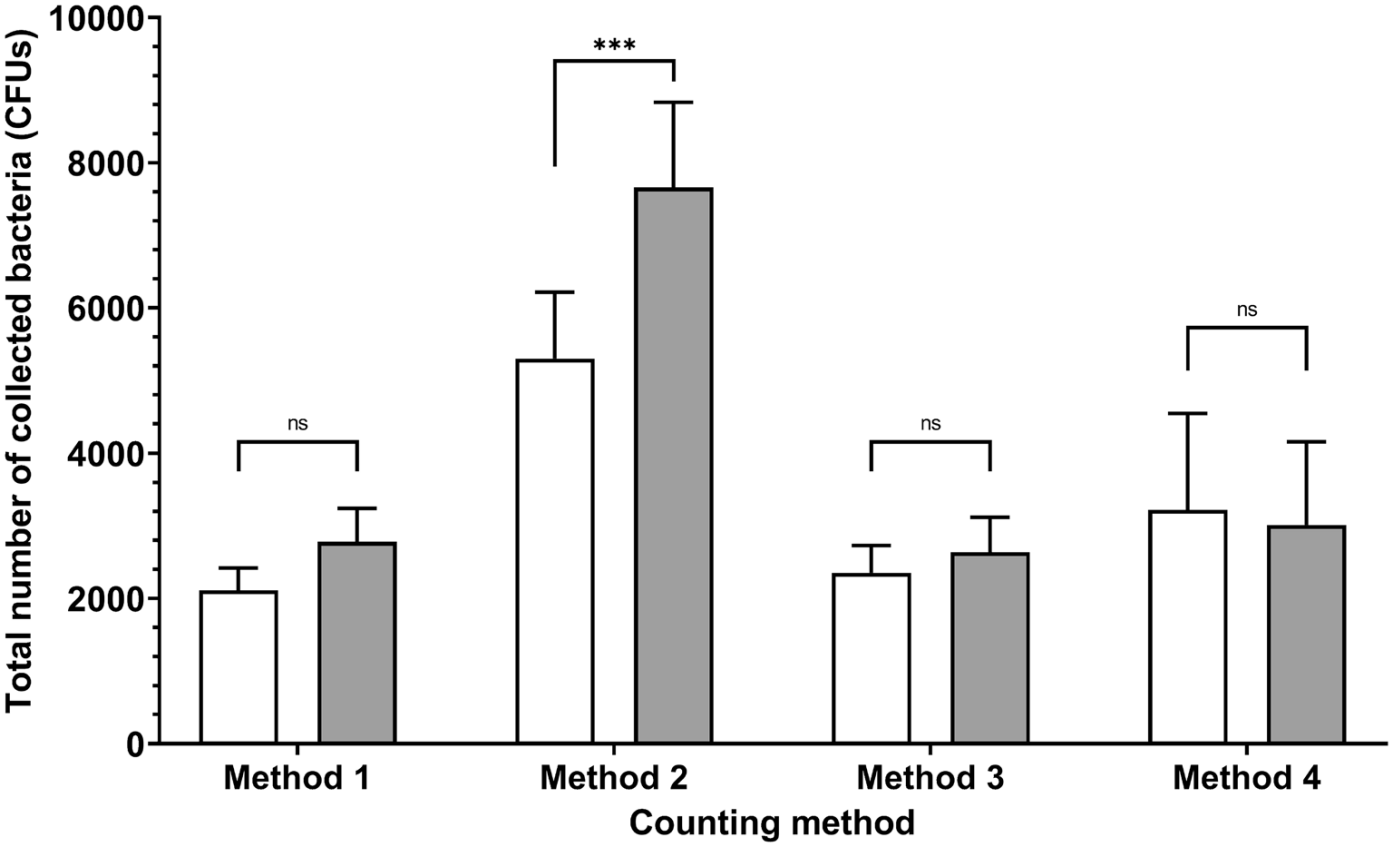
Impact of Petri dish size (90 mm vs 100 mm on the x-axis) on the total number of collected bacteria for the different colony-counting methods. The total number of collected bacteria was calculated on the six stages of the ACI for positive BFE runs (n = 4 for two different bacterial suspensions; 8 nebulizations). White bars: 90 mm petri dishes. Grey bars: 100 mm petri dishes. Method 1 corresponds to direct colony counting using an HD counter (2111 ± 310 counts for 90-mm dishes; 2781 ± 460 counts for 100-mm dishes). Method 2 corresponds to direct colony counting using an HD counter and then conversion into viable particles (5299 ± 916 counts for 90-mm dishes; 7659 ± 1177 counts for 100-mm dishes). Method 3 corresponds to visual colony counting of positive holes and then conversion (1809 ± 329 counts for 90-mm dishes; 2638 ± 480 counts for 100-mm dishes). Method 4 was used as a reference, corresponding to an approximation of the total airborne bacteria emitted by the bioaerosol nebulizer (3222 ± 1323 counts for 90-mm dishes; 3014 ± 1144 counts for 100-mm dishes). ns: not significant; *p < 0.05; **p < 0.01; ***p < 0.001.

We performed eight positive BFE runs (corresponding to 48 Petri dishes because 6 plates were analyzed via positive runs) using 100-mm Petri dishes (Fig. 5) and eight other positive BFE runs using 90-mm disposable Petri dishes. We found that (1) the bacterial challenge was similar between the positive runs using either the 100-mm or the 90-mm Petri dishes (no statistical difference in the counts for method 4), (2) use of the 90-mm Petri dish yielded similar counts to those of the 100-mm Petri dishes for methods 1 and 3, and (3) the only impact from using the 90-mm plates occurred with method 2, which was irrelevant to counting the total bacteria/viable counts from the results in Fig. 5 (Fig. 6, Supplementary Table 2).

### Effect of colony-counting methods on bioaerosol aerodynamic size

**Figure 7:**
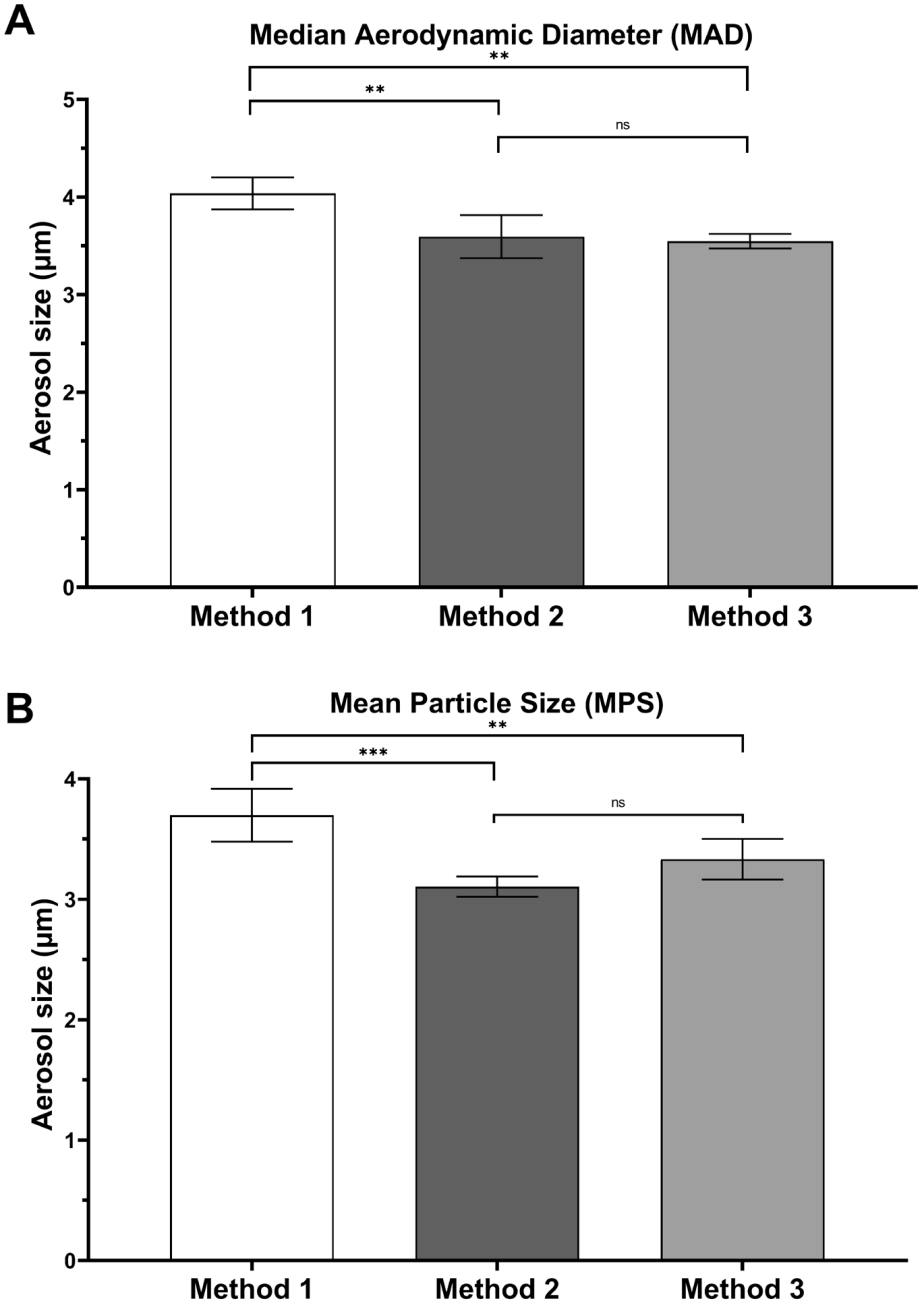
Impact of colony-counting methods on bioaerosol aerodynamic size (n = 2 for two different bacterial suspensions; 4 nebulizations/90-mm Petri dish). (**A**) Refers to the Median Aerodynamic Diameter (MAD). (**B**) Refers to the Mean Particle Size (MPS). Method 1 corresponds to direct colony counting using an HD counter (4.04 ± 0.16 µm for MAD; 3.70 ± 0.26 µm for MPS). Method 2 corresponds to direct colony counting using an HD counter and then conversion into viable particles (3.60 ± 0.22 µm for MAD; 3.11 ± 0.11 µm for MPS). Method 3 corresponds to visual colony counting of positive holes and then conversion (3.55 ± 0.08 µm for MAD; 3.33 ± 0.32 µm for MPS). ns: not significant; *p < 0.05; **p < 0.01; ***p < 0.001.

**Figure 8:**
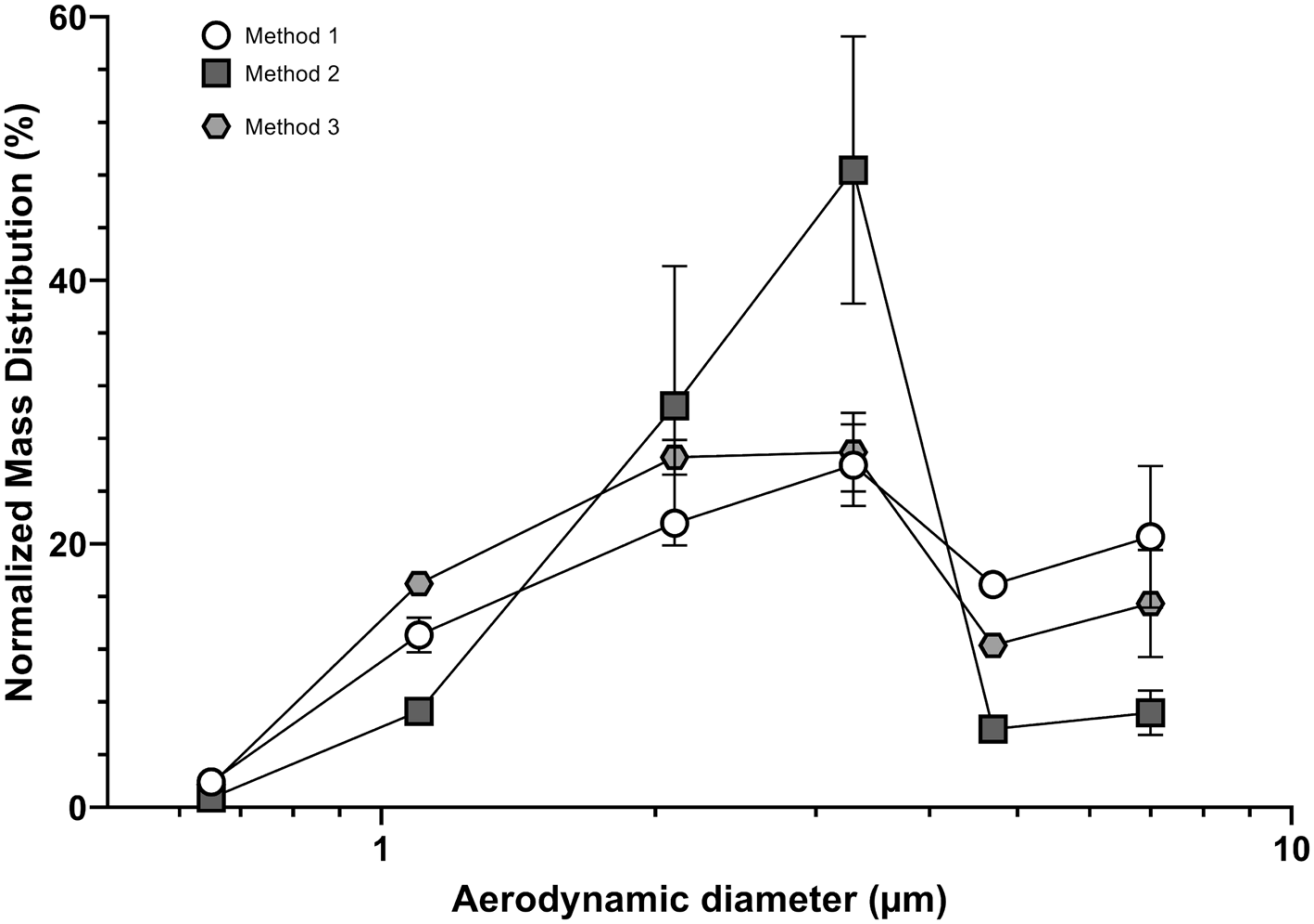
Aerosol size distribution (n = 2 for two different bacterial suspensions; 4 nebulizations/90-mm Petri dish). Method 1 (open circles) corresponds to direct colony counting using an HD counter. Method 2 (closed squares) corresponds to direct colony counting using HD counter and then conversion into viable particles. Method 3 (closed hexgons) corresponds to visual colony counting of positive holes and then conversion.

Two aerosol size parameters were calculated for each colony-counting method. Aerosol particle sizing was defined in terms of median aerodynamic diameter (MAD) or mean particle size (MPS). MAD was determined using the cumulative curve count vs. size according to European standard NF EN 13544-1. The polydispersity of the bioaerosol size distribution was calculated with the geometric standard deviation (GSD) parameter, equal to (d_84_/d_16_)^0.5^, where d84 and d16 represent the diameters of the aerosol distributions at 84% and 16%, respectively. First, compared with method 3 (the gold standard recommended by the EN 14683:219), only method 2 showed no statistical difference, and method 1 appeared to overestimate the aerosol size (Fig. 7). The MPS and MAD also led to disparate values, with the MAD value being from 0.17 to 0.5 µm higher than the MPS value (Supplementary Table 2). Figure 8 shows the aerosol size distribution of the bioaerosol emitted by the nebulizer. The bioaerosol distribution appeared polydisperse (whatever the methods used we observed a GSD range between 1.38 and 2.22, Supplementary Table 2), with particles < 1 µm (typically in the [5–15%] range depending of the method used and > 4 µm (again typically in the [5–15%] range depending of the method used), even when the major distribution mode of the aerosol distribution was centered around 2.5 µm (Fig. 8).

## Discussion

### Validation of substituting the 100-mm Petri dishes with 90-mm disposable Petri dishes

Using an appropriate Petri dish size is critical because it will alter the distance between the jet orifice and the collection surface at each stage of the ACI. Furthermore, because of the static charge generated, plastic Petri dishes can reduce the particle collection efficiency by the ACI. Using specific glass Petri dishes (~ 100 mm in diameter) may seem like a minor detail but is logistically important when performing BFE tests. Each BFE test for each mask type requires counting 48 Petri dishes (five test specimens, two positive control runs, one negative control run, and six Petri dishes for all eight of the six-stage viable ACI experiments). Considering a workload of 10 BFE tests/day, as Petri dishes should be incubated for 20–52 h according to EN 14683:2019, a stock of 960–1440 glass Petri dishes is needed with time-consuming washing and sterilization steps for hundreds of dishes per day.

To our knowledge, all laboratories that assumed high rates of testing during the period of very high demand for mask performance validation during the first half of 2020 used sterile plastic consumable Petri dishes. Some authors in the 1950s and 1980s also reported using disposable plastic Petri dishes, which are more convenient if the quantity of the medium inside the Petri dish is adjusted to maintain the correct distance between the impactor stage and the collecting surface^19,20^. Thus, our study revealed that using 90-mm Petri dishes yielded similar counts to those of the 100-mm Petri dishes for methods 1 and 3 (Fig. 6). Additionally, even if methods 1 and 3 did not statistically differ, the total bacterial count decreased when the 90-mm plates were used compared with the counts for the 100-mm plates (Fig. 6). This seems logical since the decrease in surface area for the impaction of particles inside the ACI is ~ 20% when using a 90-mm dish rather than a 100-mm dish. However, very few particles are impacted on the collection surface on the edges of the dish, which explains why this decrease did not significantly affect the total bacterial counts.

### Validation of direct colony counting using the automatic HD counter for total colony counts

Several points can be made regarding the qualitative approach proposed from the example of the dish in Fig. 2. Because the Six-Stage Viable ACI is a 400-orifice cascade impactor, the counts obtained using method 1 cannot correspond to positive holes since the maximum number of positives holes for a plate is 400. Therefore, direct counting using the automatic HD counter seems to correspond more to viable particles rather than to positive holes. In other words, use of a modern optical HD colony counter enables directly counting the viable particles on plates since several colonies can be easily observed at each impaction site coming from the same hole. Thus, if method 1 corresponds to a direct and accurate viable particle count, the result should be in good accordance with the count obtained using method 3. However, here, method 3 seemed to overestimate the viable particle count (584 for method 3 versus 479 for method 1). However, using the conversion table for positive-hole counts > 300 can also induce biases. This difference of approximately 100 viable particles corresponds to a difference of only 28 positives holes (279 for method 1 vs 307 for method 3). In other words, without the overestimation of 10% of the positive-hole counts during the visual counting corresponding to method 3, methods 1 and 3 would have yielded similar counts of viable particles. Even if nothing is concluded at this stage, a 10% overestimation error (approximately 30 positive holes) when visually counting the positive holes via method 3 is more reasonable than a 20% underestimation error (approximately 100 colonies) when directly counting using the HD colony counter via method 1. The true value may be a compromise between the true counts of viable particles from both methods.

Quantitative analysis (Fig. 5) revealed that direct counting using an automatic HD counter yielded similar results to those obtained using the gold standard procedure recommended in EN 14683:219. Methods 1 and 3 were also in good accordance with the estimation of the total airborne bacteria emitted by the bioaerosol nebulizer (method 4, Fig. 5), indicating that these two methods are reliable for calculating the true viable particle counts. However, method 1, although more expensive because it requires purchasing an HD automatic colony counter, has the advantages of being completely operator-independent, extremely fast, reproducible and highly precise. The automatic recording of an image for each Petri dish analyzed allows maintaining traceability of the results and reinterpreting them a few days or weeks later if necessary. Thus, regulatory bodies should authorize use of these modern colony counters for performing BFE tests on masks.

### Validation of direct colony counting using the automatic HD counter for aerosol size

We demonstrated that method 2 using the automatic HD counter was rigorously similar in terms of MPS and MAD calculations using method 3 (the gold standard recommended by EN 14683:2019 ). The results showed that the automatic HD counter could be a useful and reliable alternative for calculating the MPS parameters needed for BFE testing of face mask performances.

MPS and MAD yielded significantly different values, with the MPS always yielding values that were lower than those of the MAD (Fig. 7). Statistically, this difference indicates that the aerosol size did not follow a normal distribution. Standard statistics based on normal distributions tend to be unsuitable for most aerosol size distributions. Generally, lognormal distributions tend to the best fit for single source aerosol as is the case for BFE experiments. Aerosol size distribution is typically described by the mean (the first raw moment of the size distribution), the mode (the peak or maximum value of the size distribution), and the median (the middle value of a dataset, 50% of the particles are smaller than the median, and 50% are larger). The advantage of the median over the mean (often known as the average) in describing data is that the median is less skewed by a small proportion of extremely large or small values; thus, it often provides a better idea of a “typical” value. Considering the relatively high aerodynamic size polydispersity (for method 3 recommended by US and EN standard a GDS range between 1.89 and 2.22, Supplementary Table 2) yielded by bioaerosol nebulizers when conducting BFE testing (i.e., a relatively high content of particles > 4 µm and < 1 µm even if the mean mode of the distribution ranges from 2–3 µm), the MAD should be a better parameter than the MPS for a more accurate intercomparison of BFE tests using different bioaerosol sources. Finally, adding a condition on the size polydispersity of the bioaerosol emitted by the source (e.g., a limit I term of the GSD or a maximum particle content of < 1.1 µm corresponding to stage 5 of the six-stage viable ACI) may be of value because the filtration efficiency of many masks often drops from a size below 1 µm^21^.

## Conclusions

This study provided new insights regarding BFE, which is a critical performance feature of medical face masks. Our work demonstrated the following:

- Disposable 90-mm Petri dishes can be used instead of the 100-mm dishes supplied with the six-stage viable ACI.
- Automatic HD colony counters can be used to directly count viable particles on collection substrates without requiring use of the positive-hole conversion table.
- Automatic HD colony counters can be used to measure the MPS, but the positive-hole conversion table must be used to correct the data.

In summary, this important scientific input enables considering possible modernization of the outdated test methods recommended by the US and European standards on BFE measurements. Specifically, use of a modern colony counter should be regulated and permitted to avoid the drawbacks posed by visually counting the positive holes and using a conversion table to convert them into viable particles. In characterizing the particle size distribution of the bioaerosol generated, current recommendations of standards to calculate the MPS seem outdated, and the MAD seems more relevant than does the MPS for characterizing aerosol size using a lognormal distribution.

## Supplementary Information

Supplementary Information
