# Supplementary material for: New insights into the standard method of assessing bacterial filtration efficiency of medical face masks
Source: Sci Rep. 2021 Mar 15;11:5887. doi: 10.1038/s41598-021-85327-x (PMC7960701; doi:10.1038/s41598-021-85327-x)
Supplement: Supplementary file 1 — Supplementary Information [file 41598_2021_85327_MOESM1_ESM.pdf]

Supplementary file

Performances of medical face masks: new insights into the standard method of assessing bacterial filtration efficiency

POURCHEZ Jérémie[1], PEYRON Aurélien[1], MONTIGAUD Yoann[1], LAURENT Coralie[1], AUDOUX Estelle[2], LECLERC Lara[1], VERHOEVEN Paul O[2,3]

Supplementary Table 1. Positive-hole conversion table from the instruction manual of the six-stage viable ACI.

r, positive-hole count; P, corresponding corrected viable particle count.

r	*P*	*r*	*P*	*r*	*P*	*r*	*P*	*r*	*P*	*r*	*P*	*r*	*P*	*r*	*P*	*r*	*P*	*r*	*P*
1	1	41	43	81	91	121	144	161	206	201	279	241	369	281	485	321	649	361	931
2	2	42	44	82	92	122	146	162	208	202	281	242	372	282	488	322	654	362	942
3	3	43	45	83	93	123	147	163	208	203	283	243	374	283	492	323	659	363	952
4	4	44	47	84	94	124	148	164	211	204	285	244	377	284	495	324	664	364	963
5	5	45	48	85	96	125	150	165	213	205	287	245	379	285	499	325	670	365	974
6	6	46	49	86	97	126	151	166	214	206	289	246	382	286	502	326	675	366	986
7	7	47	50	87	98	127	153	167	216	207	292	247	384	287	506	327	680	367	998
8	8	48	51	88	99	128	154	168	218	208	294	248	387	288	508	328	686	368	1010
9	9	49	52	89	101	129	156	169	220	209	296	249	390	289	513	329	692	369	1023
10	10	50	53	90	102	130	157	170	221	210	298	250	392	290	516	330	697	370	1036
11	11	51	55	91	103	131	159	171	223	211	300	251	395	291	520	331	703	371	1050
12	12	52	56	92	105	132	160	172	225	212	302	252	398	292	524	332	709	372	1064
13	13	53	57	93	106	133	162	173	227	213	304	253	400	293	527	333	715	373	1078
14	14	54	58	94	107	134	163	174	228	214	306	254	403	294	531	334	721	374	1093
15	15	55	59	95	108	135	165	175	230	215	308	255	406	295	535	335	727	375	1109
16	16	56	60	96	110	136	166	176	232	216	311	256	409	296	539	336	733	376	1125
17	17	57	61	97	111	137	168	177	234	217	313	257	411	297	543	337	739	377	1142
18	18	58	63	98	112	138	169	178	236	218	315	258	414	298	547	338	746	378	1160
19	19	59	64	99	114	139	171	179	237	219	317	259	417	299	551	339	752	379	1179
20	21	60	65	100	115	140	172	180	239	220	319	260	420	300	555	340	759	380	1198
21	22	61	66	101	116	141	174	181	241	221	322	261	423	301	559	341	766	381	1219
22	23	62	67	102	118	142	175	182	243	222	324	262	426	302	563	342	772	382	1241
23	24	63	69	103	119	143	177	183	245	223	326	263	429	303	567	343	779	383	1263
24	25	64	70	104	120	144	179	184	246	224	328	264	432	304	571	344	786	384	1288
25	26	65	71	105	122	145	180	185	248	225	231	265	434	305	575	345	793	385	1314
26	27	66	72	106	123	146	182	186	250	226	333	266	437	306	579	346	801	386	1341
27	28	67	73	107	125	147	183	187	252	227	335	267	440	307	584	347	808	387	1371
28	29	68	75	108	126	148	185	188	254	228	338	268	443	308	588	348	816	388	1403
29	30	69	76	109	127	149	186	189	256	229	340	269	447	309	592	349	824	389	1438
30	31	70	77	110	129	150	188	190	258	230	342	270	450	310	597	350	832	390	1476
31	32	71	78	111	130	151	190	191	260	231	345	271	453	311	601	351	840	391	1518
32	33	72	79	112	131	152	191	192	262	232	347	272	456	312	606	352	848	392	1565
33	34	73	81	113	133	153	193	193	263	233	349	273	459	313	610	353	857	393	1619
34	36	74	82	114	134	154	194	194	265	234	352	274	462	314	615	354	865	394	1681
35	37	75	83	115	136	155	196	195	267	235	354	275	465	315	620	355	874	395	1754
36	38	76	84	116	137	156	198	196	269	236	357	276	468	316	624	356	883	396	1844
37	39	77	86	117	138	157	199	197	271	237	359	277	472	317	629	357	892	397	1961
38	40	78	87	118	140	158	201	198	273	238	362	278	475	318	634	358	902	398	2127
39	41	79	88	119	141	159	203	199	275	239	364	279	478	319	639	359	911	399	2427
40	42	80	89	120	143	160	204	200	277	240	367	280	482	320	644	360	921	400	*

Supplementary Table 2. Data for comparing the bacterial counting methods corresponding to Figures 5, 6, 7 and 8.

		90-mm Petri dish			100-mm Petri dish		
		**Method 1**	**Method 2**	**Method 3**	**Method 1**	**Method 2**	**Method 3**
**Aerosol size (µm)**	**MPS (n=4)**	3.70 ± 0.22	3.10 ± 0.08	3.31 ± 0.16	4.06 ± 0.13	3.04 ± 0.13	3.87 ± 0.10
	**MAD (n=4)**	4.04 ± 0.16	3.60 ± 0.22	3.55 ± 0.08	4.23 ± 0.15	3.29 ± 0.18	3.87 ± 0.10
**GSD of the aerosol size distribution (n=4)**		1.88 ± 0.07	1.38 ± 0.02	1.89 ± 0.92	2.12 ± 0.15	1.57 ± 0.14	2.22 ± 0.20
**Colonies counted (CFUs; n=8)**		2111 ± 310	5299 ± 916	2351 ± 377	2781 ± 460	7659 ± 1177	2638 ± 480
